# Effects of Pin1 Loss in *Hdh^Q111^* Knock-in Mice

**DOI:** 10.3389/fncel.2016.00110

**Published:** 2016-05-02

**Authors:** Elena Agostoni, Silvia Michelazzi, Marta Maurutto, Alisia Carnemolla, Yari Ciani, Paolo Vatta, Paola Roncaglia, Silvia Zucchelli, Giampiero Leanza, Fiamma Mantovani, Stefano Gustincich, Claudio Santoro, Silvano Piazza, Giannino Del Sal, Francesca Persichetti

**Affiliations:** ^1^International School for Advanced Studies (SISSA), Area of NeuroscienceTrieste, Italy; ^2^Laboratorio Nazionale del Consorzio Interuniversitario per le Biotecnologie (LNCIB), Area Science ParkTrieste, Italy; ^3^Department of Life Sciences, University of TriesteTrieste, Italy; ^4^Department of Health Sciences, University of Piemonte OrientaleNovara, Italy; ^5^Department of Neuroscience and Brain Technologies, Italian Institute of TechnologyGenova, Italy

**Keywords:** Huntington’s disease, Pin1, DNA damage response, gliosis, neuronal intranuclear inclusions

## Abstract

Huntington’s disease (HD) is a fatal, dominantly inherited, neurodegenerative disorder due to a pathological expansion of the CAG repeat in the coding region of the *HTT* gene. In the quest for understanding the molecular basis of neurodegeneration, we have previously demonstrated that the prolyl isomerase Pin1 plays a crucial role in mediating p53-dependent apoptosis triggered by mutant huntingtin (mHtt) *in vitro*. To assess the effects of the lack of Pin1 *in vivo*, we have bred Pin1 knock-out mice with *Hdh^Q111^* knock-in mice, a genetically precise model of HD. We show that Pin1 genetic ablation modifies a portion of *Hdh^Q111^* phenotypes in a time-dependent fashion. As an early event, Pin1 activity reduces the DNA damage response (DDR). In midlife mice, by taking advantage of next-generation sequencing technology, we show that Pin1 activity modulates a portion of the alterations triggered by mHtt, extending the role of Pin1 to two additional *Hdh^Q111^* phenotypes: the unbalance in the “synthesis/concentration of hormones”, as well as the alteration of “Wnt/β-catenin signaling”. In aging animals, Pin1 significantly increases the number of mHtt-positive nuclear inclusions while it reduces gliosis. In summary, this work provides further support for a role of Pin1 in HD pathogenesis.

## Introduction

Huntington’s disease (HD) is a dominantly inherited neurodegenerative disorder, characterized by motor impairment and cognitive decline. Neuropathologically, HD presents selective neuronal loss in the striatum associated to astrocytic gliosis that increases with disease progression (Vonsattel et al., 1985). In the later stage of the disease, pathologic alterations have been described in other brain regions, including cerebral cortex, thalamus, and subthalamic nucleus (Vonsattel and DiFiglia, 1998).

The HD mutation alters a polymorphic CAG trinucleotide repeat in the first exon of *HTT*, the gene encoding for huntingtin (The Huntington’s Disease Collaborative Research Group, 1993). The number of CAG repeats varies between 6 and 35 units on normal chromosomes, whereas on HD chromosomes the repeat is expanded above the pathological threshold of 36 CAGs. This leads to the synthesis of a mutant protein containing an expanded polyglutamine tract at the N-terminus that confers to the protein the tendency to aggregate. Neuronal intranuclear inclusions (NIIs) and neuropil aggregates that stain positive for huntingtin are a clear histopathological marker of the disease (DiFiglia et al., 1997; Gutekunst et al., 1999).

Mutant huntingtin (mHtt) triggers a large plethora of cellular dysfunctions, as reviewed by Zuccato et al. (2010) and Labbadia and Morimoto (2013). Among them, there is transcriptional deregulation as well as induction of nucleolar and endoplasmic reticulum stress (Sugars and Rubinsztein, 2003; Cha, 2007; Carnemolla et al., 2009; Lee et al., 2014). mHtt expression is also associated with extensive Deoxyribonucleic acid (DNA) lesions and the activation of the ATM/ATR DNA damage response (DDR) pathway with consequent phosphorylation of H2AX at the damaged sites and stabilization of p53, which in turn mediates mitochondrial dysfunction and cytotoxicity (Bae et al., 2005; Anne et al., 2007; Illuzzi et al., 2009).

Recently, a genome-scale RNAi screen to search for pathways interfering with mHtt toxicity has identified peptidyl-prolyl cis/trans isomerase NIMA-interacting 1 (Pin1; Miller et al., 2012). siRNA reduction of Pin1 was found to decrease caspase-3 activation induced by the expression of a truncated N-terminal fragment of mHtt in HEK293T cells. Similarly, in mutant ST*Hdh^Q111^* striatal cell line, knock-down of Pin1 reduced caspase activation triggered by serum deprivation to a level comparable to controls. Furthermore, the motor performance defect in a *Drosophila* HD model was significantly rescued upon decreased expression of the Pin1 homolog *dodo* (Miller et al., 2012). Intriguingly, juglone, a potent Pin1 inhibitor, was previously found to reverse the abnormal cellular localization of full-length mHtt in ST*Hdh^Q111^* striatal cells (Wang et al., 2005).

Phosphorylation—dependent prolyl isomerization triggered by Pin1 represents an essential mechanism in modulating many cellular processes. Pin1 is highly expressed in the brain where it plays a critical role in healthy aging and prevention of age-related neurodegeneration (Liou et al., 2003; Sorrentino et al., 2014). Detailed molecular analyses in mice and cells led to the findings that in Alzheimer’s disease Pin1 regulates amyloid precursor protein (APP) processing and amyloid-beta production (Pastorino et al., 2006) while it is required for Tau dephosphorylation and correct neurofibrillary organization (Liou et al., 2003). Furthermore, in Parkinson’s disease Pin1 facilitates formation of alpha-synuclein inclusions by regulating its binding partner synphilin (Ryo et al., 2006).

We have previously shown that in cell culture and in mouse brain Pin1 activity is required for p53 activation in response to mHtt (Grison et al., 2011). In HD cellular models and human post-mortem brains, p53 is phosphorylated on Ser46, a modification that triggers the interaction with Pin1 and activates p53 apoptotic functions. As a consequence, the genetic ablation of Pin1 prevents the expression of p53 apoptotic target genes in the striatum of *Hdh^Q111^* mice (Grison et al., 2011).

*Hdh^Q111^* knock-in mice (White et al., 1997) display molecular and behavioral phenotypes that recapitulate, in a time-dependent fashion, many features of the human disease (Fossale et al., 2002; Wheeler et al., 2002; Gines et al., 2003; Lloret et al., 2006; Lynch et al., 2007; Carnemolla et al., 2009; Giralt et al., 2012; Hölter et al., 2013). In the first months of life, initial molecular changes (here referred to as “early phenotypes”) include the activation of DDR and the induction of ribosome biogenesis regulator 1 (*Rrs1*) expression, a nucleolar protein involved in rRNA biogenesis and endoplasmic reticulum stress. At a later age (“intermediate phenotypes”), mHtt alters the expression of several pathways and genes with a plethora of different functions. In aging animals (“late phenotypes”), NIIs decorate the large majority of striatal neurons while gliosis infiltrate the nervous system as evidenced in human post-mortem brains.

Since Pin1 activity is involved in DDR and formation of protein aggregates, here we have taken advantage of *Hdh^Q111^* mice in a Pin1 knock-out genetic background (Grison et al., 2011) to prove that Pin1 modulates multiple *Hdh^Q111^* phenotypes throughout the lifespan of the rodent.

## Materials and Methods

### Mice Strain

Heterozygous (*Hdh^Q7/Q111^*) knock-in mice in C57BL/6 background (Lloret et al., 2006) were provided by M. MacDonald (Massachusetts General Hospital, Boston, MA, USA). Pin1 knock-out mice in C57BL/6 background (Atchison et al., 2003) were provided by A. Means (Duke University, Durham, NC, USA). *Hdh^Q7/Q111^* and *Pin1^WT/KO^* mice were crossed to generate double heterozygous mice. These mice were then crossed to generate littermates of different genotypes. Mice used in this study were *Hdh^Q7/Q7^*, *Hdh^Q111/Q111^* (3.5 and 12 months) or *Hdh^Q7/Q111^* (24 months) either wild-type (*Pin1^WT/WT^*) or homozygous knock-out (*Pin1^KO/KO^*) for Pin1. Genotyping for both loci was performed by PCR on tail DNA as described previously (White et al., 1997; Atchison et al., 2003). Mice were balanced with respect to gender except for the gene expression profiling experiments where only female mice were used. *Hdh^Q111^*:*Pin1^KO^* mice appear healthy, do not display any gross physical or behavioral abnormalities and have a normal life span. Animal care, handling and subsequent procedures were performed in accordance with the European Community Council Directive of November 24, 1986 (86/609EEC) and following SISSA Ethical Committee permissions.

### RNA Isolation and Real Time PCR

Total RNA was extracted from mouse striata using TRIzol^®^ reagent (Invitrogen) according to manufacturer’s instructions. RNA was quantified (Qubit, Life Technologies) and quality was monitored using a Bioanalyzer (Agilent Technologies).

*Rrs1* RT-qPCR was performed with CFX96 Touch^TM^ Real-Time PCR instrument (Bio-Rad), using the iQ SYBR Green Supermix (Bio-Rad) as previously described (Carnemolla et al., 2009). Normalized expression values were calculated using *β*-actin as the endogenous control.

### RNA-Seq and Data Processing

RNAseq was performed by IGA Technology Services, Udine, Italy. Libraries were prepared using the TruSeq RNA Kit (Illumina) and sequenced with a HiSeq2000 sequencer (Illumina) to generate 50bp single reads. For each library, more than 40 million of reads were obtained.

Quality control of RNA-seq reads was performed using FastQC^1^. Genome mapping was carried out with STAR aligner (v2.3.0, ENSEMBL.mus_musculus.release-75 as reference genome, Dobin et al., 2013). Mapped reads were counted with HTSeq (v0.6,^2^ Anders et al., 2015). Differential gene expression analysis was performed using the gene raw counts, within the R/Bioconductor edgeR package (Robinson et al., 2010). The bioinformatic pipeline was customized to: (1) estimate the dispersion parameter for each library using the biological group dispersion; (2) identify differentially expressed genes between the animal models *(Hdh^Q111^* vs. *Hdh^Q7^* in *Pin1^WT^* background, *Pin1^WT^* vs. *Pin1^KO^* in *Hdh^Q111^* background); (3) consider log2 (fold-change) ≥0.7 as threshold; and (4) adjust the *P*-value for multiple testing using the Benjamini-Hochberg correction with a false discovery rate (FDR) ≤0.05. Functional annotation, category and pathway analysis of the differential gene lists was carried out using the R/Bioconductor ClusterProfiler (Yu et al., 2012) package and Ingenuity Pathway Analysis tool (IPA; QIAGEN Silicon Valley, Redwood City, CA, USA^3^). For ClusterProfiler we calculated the enrichment value (*p* < 0.05, additional parameters as standard) for biological process, molecular function and cellular component. For IPA, transcripts were associated with biological functions/transcriptional regulators in the Ingenuity Knowledge Base. Right-tailed Fisher’s exact test was used to calculate a *p*-value determining the probability that each biological function/transcriptional regulator assigned to that data set is due to chance alone. All sequencing data were submitted to the Gene Expression Omnibus (GEO; accession number GSE64478).

### Western Blot

Total protein lysates from mouse striatum were extracted using TRIzol^®^ reagent (Invitrogen) according to manufacturer’s instructions, after RNA extraction. Ten microgram (10 μg) of protein lysates were analyzed by western blot. Primary antibodies were: anti γ-H2AX (1:1000, Millipore), anti-β-actin (1:5000, A1978, Sigma) and anti-Pin1 (1:2000, G-8, Santa Cruz Biotech).

Relative quantification of protein bands from western blot scans was performed with ImageJ Software.

### Immunohistochemistry

Animals were transcardially perfused with 4% paraformaldehyde in Phosphate-Buffered Saline (PBS) solution (pH 7.4). The brain was rapidly dissected out, post-fixed o/n at 4°C in the same fixative and then kept at 4°C in 30% sucrose/PBS until it had sunk. Using a vibratome (Vibratome 1000 Plus Sectioning System, St Louis, MO, USA), coronal sections of 40 μm thickness were cut throughout the striatum (−3 mm to +3 mm from Bregma) and every 10th sections samples were processed for immunohistochemistry. To block endogenous peroxidase free-floating sections were treated for 10 min with Tris-Buffered Saline (TBS) solution containing 10% methanol and 3% H_2_O_2_. After washing in TBS, sections were incubated for 1 h in blocking solution (1% BSA, 10% FCS, 1% fish gelatin) and then o/n at 4°C with the primary antibodies in TBS containing 1% BSA, 0.3% Triton X-100 and 0.1% fish gelatin. Sections were washed and incubated for 1 h with secondary anti-mouse Alexa-488 conjugated antibody (1:2000). Images were captured using a Leica TCS SP2 confocal microscope.

Primary antibodies were: EM48 anti-huntingtin (1:100, MAB5374, Chemicon), anti-glial fibrillary acidic protein (GFAP; 1:1000, G6171, Sigma) and anti-γH2AX (1:500, 05–636, Millipore).

### Huntingtin Inclusions and GFAP Fluorescence Quantification

Quantification of nuclear inclusions in 24 month old mice was performed as follow. Three serial coronal sections (0.78–0 mm from bregma) were chosen for each mouse. In each section two areas of the striatum (375 μm^2^ each), one dorsal and one ventral, were identified using a 40× oil objective. Confocal photomicrograhs of six *Z*-sections (5 μm) of the selected areas were taken and the total number of nuclear inclusions was counted in the *Z-stack* images using eCELLence Software (Glance Vision Technologies, Trieste, Italy). The total number of inclusions was counted per mouse and the arithmetic mean of four mice for each genotype was calculated and expressed as number of NI/100 μm^2^. The same method was applied to evaluate the number of inclusions in piriform cortex and olfactory tubercles regions (40× objective, one area of 375 μm^2^ per section). GFAP fluorescence intensity was evaluated in the same striatal areas described above with a 20× objective (one area of 750 μm^2^ per section, three sections per mice, four mice per genotype) using the Leica Confocal Software Histogram Quantification Tool. Pixel mean fluorescence intensity (expressed in arbitrary units) of the selected area was normalized to background fluorescence (region without cells). A paired two-tailed Student’s *t*-test (Microsoft Excel Software) was used for statistical analysis. Values with *p* < 0.05 were considered significant.

### Statistical Analysis

Depending on the number of groups within the data set, data were analyzed using either the Student’s *t-*test (two groups) or one-way ANOVA with Tukey’s HSD *post hoc* test (three groups). The *p* values < 0.05 were considered statistically significant. Calculated means and standard errors were plotted using the graph tool of Microsoft Excel.

## Results

### Effects of Loss of Pin1 on *Hdh^Q111^* “Early Phenotypes”

A number of alterations have been identified so far as early events in *Hdh^Q111^* mice. Among them we have studied the effects of Pin1 activity on DDR and *Rrs1* induction. These phenotypes were assessed by analyzing homozygous *Hdh^Q111^* mice that were either wild-type (*Hdh^Q111^*:*Pin1^WT^*) or knock-out for Pin1 (*Hdh^Q111^*:*Pin1^KO^*) at 3.5 months of age.

#### Pin1 Modulates DDR Induced by mHtt in Mouse Striatum

**Figure 1:**
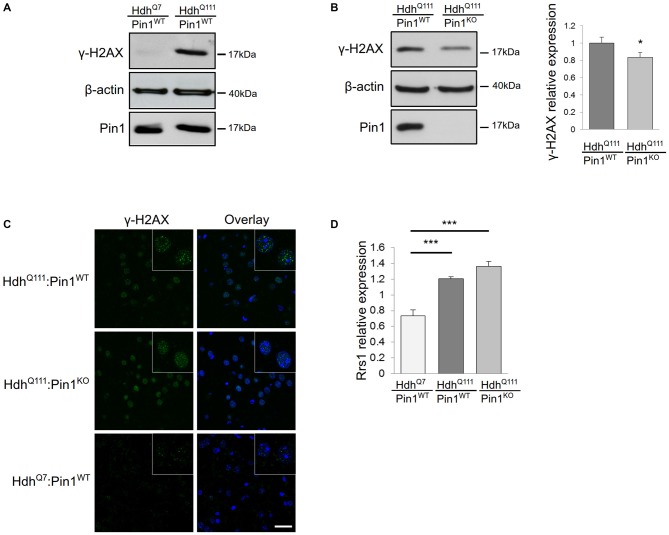
**Consequences of Pin1 depletion on *Hdh^Q111^* “early phenotypes”. (A–C)** Pin1 modulates mutant huntingtin (mHtt)-induced DNA damage. **(A)** Representative western blot showing γH2AX protein levels in lysates from striatum of homozygous *Hdh^Q111^* and *Hdh^Q7^* mice at 3.5 months of age. β-actin antibody was used as control of sample loading. **(B)** Representative western blot showing comparison of γH2AX protein levels in striatum of *Hdh^Q111^* mice on wild-type (*n* = 6) or knock-out (*n* = 6) Pin1 genetic backgrounds at 3.5 months of age. β-actin was used as a loading control. The histogram on the right shows relative quantifications of γH2AX signals between the two genotypes. Histogram bars represent mean ± standard error (*Two tail paired *t*-test *p* < 0.05). **(C)** Comparison of immunofluorescence staining of γH2AX foci in coronal brain sections from striatum of 3.5 months mice of the indicated genotypes. The nuclear staining with 4′,6-diamidino-2-phenylindole (DAPI) is shown in blue. Scale bar, 30 μm. Inset shows γH2AX foci at higher magnification. Scale bar, 10 μm. **(D)** Rrs1 mRNA induction is not modulated by Pin1. RT-qPCR analysis was performed to detect levels of *Rrs1* relative to β-actin mRNAs in striatum of *Hdh^Q7^* (*n* = 6) and homozygous *Hdh^Q111^* mice on wild type (*n* = 6) or knock out (*n* = 6) Pin1 genetic background at 3.5 months of age. A significant increase of *Rrs1* mRNA is found in *Hdh^Q111^*:*Pin1^WT^* and *HdhQ^111^*:*Pin1^KO^* mice compared to *Hdh^Q7^*:*Pin1^WT^* mice. No significant difference in the level of *Rrs1* mRNA is detected in *Hdh^Q111^*:*Pin1^WT^* compared to *Hdh^Q111^*:*Pin1^KO^* mice. Histogram bars represent mean ± standard error (***one-way ANOVA Tukey HSD *post hoc* test, *p* < 0.001).

Many studies in cell lines, transgenic mouse models and HD post-mortem brains have shown that mHtt can induce DNA damage (Giuliano et al., 2003; Anne et al., 2007; Stack et al., 2008; Illuzzi et al., 2009). Importantly, Pin1 has also been linked to DDR, particularly in the regulation of double strand break repair (Steger et al., 2013). Therefore, we compared the level of DNA damage in *Hdh^Q111^:Pin1^WT^* and *Hdh^Q111^*:*Pin1^KO^* mice. For this purpose we assayed the DNA damage marker γH2AX by immunofluorescence and western blot analysis (Figures 1A–C). Phosphorylation of the histone variant H2AX, forming γH2AX-labeled foci, represents the earliest visible response to the induction of DNA double-strand breaks. As expected, protein extracts from striatum of *Hdh^Q111^* mice showed high levels of phosphorylated H2AX compared to wild-type littermates, supporting the presence of DNA damage in neurons of 3.5 month old *Hdh^Q111^* mice (Figure 1A). Interestingly, the lack of Pin1 expression decreased the intensity of γH2AX signal (Figures 1B–C). Western blot quantification of γH2AX relative to β-actin indicated that, in absence of Pin1, the expression of phosphorylated H2AX is significantly reduced by about 20% (Figure 1B). These results suggest that Pin1 modulates striatal DNA damage induced by mHtt.

#### Rrs1 Induction by mHtt is not Modulated by Pin1

We have previously demonstrated that *Rrs1* mRNA expression is increased in post-mortem brain of HD patients (Fossale et al., 2002). Rrs1 is a nucleolar protein involved in rRNA biogenesis and a component of the endoplasmic reticulum stress response (Carnemolla et al., 2009). Its induction is one of the earliest events occurring in *Hdh^Q111^* mice that persist throughout the life span of the rodent (Fossale et al., 2002; Carnemolla et al., 2009). To test whether Pin1 could modulate *Rrs1* expression in *Hdh^Q111^* mice, we compared the expression level of *Rrs1* by RT-qPCR in *HdhQ^111^*:*Pin1^WT^* and *Hdh^Q111^*:*Pin1^KO^* at 3.5 months of age. As expected, *Rrs1* is induced by 1.6 fold in *Hdh^Q111^* compared to wild type littermates in a Pin1 wild type background (Figure 1D). No significant difference was detectable between *Hdh^Q111^*:*Pin1^WT^* and *Hdh^Q111^*:*Pin1^KO^* mice.

In summary these results suggest that Pin1 may modify some but not all of the “early phenotypes” selected for assessment in *Hdh^Q111^* mice.

### Effects of Lack of Pin1 on *Hdh^Q111^* “Intermediate Phenotypes”

A plethora of genes and pathways are known to be altered in the striatum of midlife HD mouse models. To estimate the portion of *Hdh^Q111^* “intermediate phenotypes” that requires a functional Pin1, we decided to carry out an unbiased transcriptome-wide approach by taking advantage of next generation sequencing technology.

We thus carried out a search for differentially expressed transcripts in the following genotypes: *Hdh^Q7/Q7^:Pin1^WT/WT^*, *Hdh^Q111/Q111^*:*Pin1^WT/WT^*, *Hdh^Q7/Q7^*:*Pin1^KO/KO^* and *Hdh^Q111/Q111^*:*Pin1^KO/KO^*. RNAs from the striatum of three mice of 12 months of age were purified for each genotype to carry out gene expression profiling with next-generation sequencing technology with the MiSeq Illumina platform and analyzed following standard best practices.

**Figure 2:**
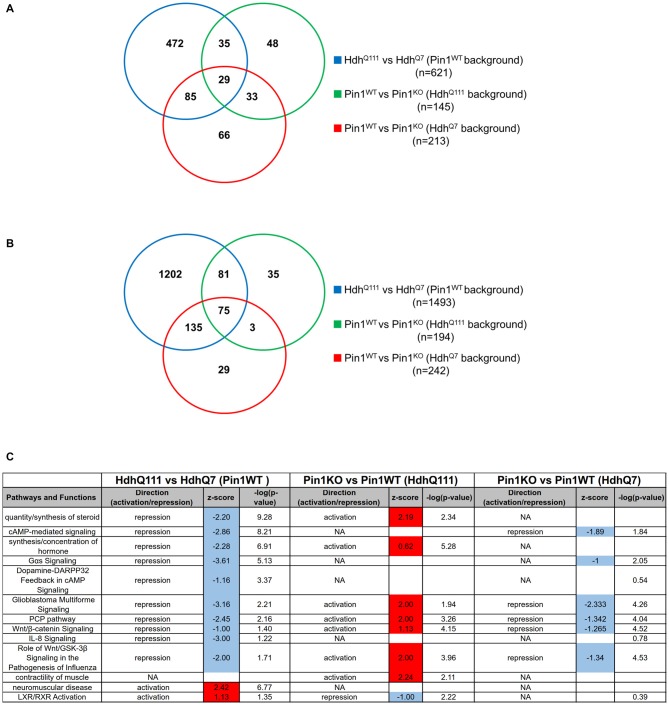
**Transcriptome functional analysis in the striatum of midlife mice suggests that loss of Pin1 reverts biological functions altered by mutant huntingtin (mHtt). (A)** Venn diagram showing the overlap of differentially expressed gene lists among the comparisons of the indicated genotypes (Supplementary Table T2). **(B)** Venn diagram showing the overlap of the biological terms among the comparisons of the indicated genotypes (Supplementary Table T4). **(C)** Table showing the predicted activation and repression of different biological terms using Ingenuity Pathway Analysis (IPA). Activation *Z*-score and –log (*p*-value) are shown. Activation *Z*-score makes predictions about potential regulators by using information about the direction of gene regulation. Red or blue box correspond to activation or repression prediction of the biological pathways.

First we compared *Hdh^Q111^* with *Hdh^Q7^* mice on a Pin1 wild-type background to describe differences in gene expression triggered by mHtt. Overall we obtained a list of 621 genes significantly altered by mHtt (Figure 2A, Supplementary Table T1). We then asked whether lack of a functional Pin1 modifies the expression pattern of *Hdh^Q111^* mice identifying 145 genes as differentially regulated between Pin1 wild-type and knock-out mice on a mHtt background.

Interestingly, 44% of them (64/145, overlap *p*-value = 10^−64^) were commonly targeted by mHtt in a wild-type genotype for Pin1 suggesting that Pin1 may act on pathways shared with mHtt. In the same stage and tissue, loss of Pin1 in a wild-type Htt background was altering the expression of 213 genes.

In summary, 29 genes (out of 64) were differentially regulated in all three comparisons while 35 of the genes altered in *Hdh^Q111^* mice were changed by loss of Pin1 activity exclusively in mHtt but not in *Hdh^Q7^* mice (Figure 2A, Supplementary Tables T1, T2).

We then analyzed the enrichment for Gene Ontology biological terms in the three differentially expressed gene lists using ClusterProfiler (Supplementary Table T3). While mHtt changed the expression of genes associated to 1493 biological terms, the effects of Pin1 loss on mHtt were described with 194 terms. Importantly, 156 of them were common in the two lists (Figure 2B). Loss of Pin1 in a wild-type Htt background was involving 242 biological terms.

In summary, loss of Pin1 in *Hdh^Q111^* background affected only 10% (156/1493) of the biological terms targeted by mHtt alone. These represented 80% (156/194) of all the biological terms associated to loss of Pin1 in mHtt background. Seventy five (out of 156) were common among all three comparisons and 81 were part of a Pin1-dependent gene expression pattern exclusively triggered by mHtt (Supplementary Table T4).

Since this type of analysis does not allow the prediction of the activation/inhibition status for each biological term, we took advantage of the IPA Downstream Effects Analysis tool to identify biological functions that are expected to increase or decrease according to gene expression changes. By comparing the gene lists in Supplementary Table T1 we observed enriched biological functions in opposite directions (activation/inhibition) when induced by mHtt on a Pin1 wild-type background compared to loss of Pin1 in a mHtt background (Pearson’s Correlation = 0.69, *p*-value = 10^−10^; Figure 2C). Among them, the biological functions “quantity/synthesis of steroid” and “synthesis/concentration of hormone” triggered by mHtt were reverted by loss of Pin1. These two pathways were not significantly affected in Pin1 knock-out mice in a wild-type Htt background. Loss of Pin1 in *Hdh^Q111^* mice was able to counteract changes in “Wnt/β-catenin signaling”, “PCP pathway” and “Role of Wnt/GSK-3β signaling in the pathogenesis of influenza” as triggered by mHtt. These pathways were also altered in Pin1 knock-out mice that were wild-type for Htt (Figure 2C). In Supplementary Table T5 gene lists associated to each pathways and biological functions are shown.

### Effects of Lack of Pin1 on *Hdh^Q111^* “Late Phenotypes”

Aging *Hdh^Q111^* mice recapitulate two neuropathological hallmarks of human HD post-mortem brains: the presence of NIIs in striatal neurons and the occurrence of massive gliosis. Therefore we investigate these phenotypes in heterozygous *Hdh^Q111^* mice either wild-type (*Hdh^Q111^*:*Pin1^WT^*) or knock-out (*Hdh^Q111^*:*Pin1^KO^*) for Pin1 at 24 months of age.

#### Mutant Huntingtin NIIs in Mouse Striatum are Increased in the Absence of Pin1

**Figure 3:**
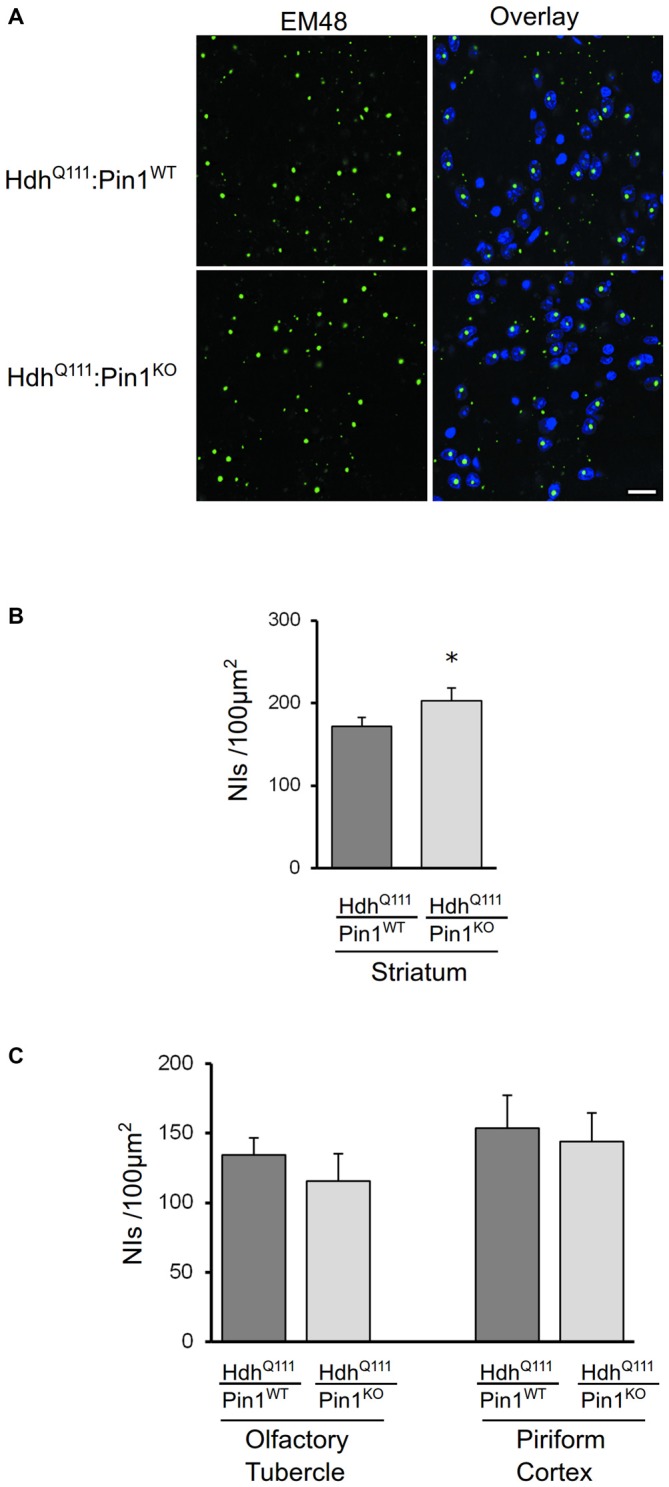
**The number of mHtt intranuclear inclusions in aged mice is modulated by Pin1 in striatum but not in olfactory tubercle and piriform cortex. (A)** Representative confocal images of EM48 positive neuronal intranuclear inclusions (NIIs) from striatal sections of 24 months mice (genotypes as indicated). The nuclear staining with 4′, 6-diamidino-2-phenylindole (DAPI) is shown in blue. Scale bar, 20 μm. **(B)** The number of NIIs in striatum of heterozygous *Hdh^Q111^* mice lacking of Pin1 is significantly increased compared to *Hdh^Q111^*:*Pin1^WT^* mice. The histogram shows quantification of EM48-positive inclusions in striatum of *Hdh^Q111^* mice on wild-type or knock-out Pin1 genotype. Histogram bars represent mean ± standard error of the number of inclusions per 100 μm^2^, three sections per mouse and four mice per genotype. (*Two tail paired *t*-test *p* < 0.05). **(C)** Histograms showing quantification of EM48-positive inclusions in olfactory tubercle (left) and piriform cortex (right) of *Hdh^Q111^* mice on wild-type (*n* = 4) or knock-out (*n* = 4) Pin1 genotype at 24 months of age. No significant differences are observed in these brain regions between the two genotypes.

NIIs of amino-terminal fragments of mHtt are a well-known histopathological marker of HD (DiFiglia et al., 1997; Gutekunst et al., 1999). Pin1 has been previously found to modulate protein aggregate formation in other neurodegenerative diseases (Lu et al., 1999; Ryo et al., 2006; Kesavapany et al., 2007). Coronal sections of mouse brains were immunostained with EM48 antibody that selectively labels mHtt. As shown in Figure 3A, mHtt was detectable in striatum of both genotypes as nuclear inclusions. Interestingly, the number of NIIs was significantly increased in *Hdh^Q111^* mice lacking Pin1 compared to *Hdh^Q111^*:*Pin1^WT^* mice (Figure 3B). At 24 months of age, NIIs were also detectable in brain area other than the striatum including the olfactory tuberculus and piriform cortex (Supplementary Figure S1). However, no differences were detected in these regions between *Hdh^Q111^*:*Pin1^WT^* and *Hdh^Q111^*:*Pin1^KO^* mice (Figure 3C). In summary, Pin1 loss increases NIIs formation selectively in the striatum proving that Pin1 plays a role in NIIs formation *in vivo*.

#### Astrocytic Gliosis in Striatum of *Hdh^Q111^* Mice is Reduced in the Absence of Pin1

**Figure 4:**
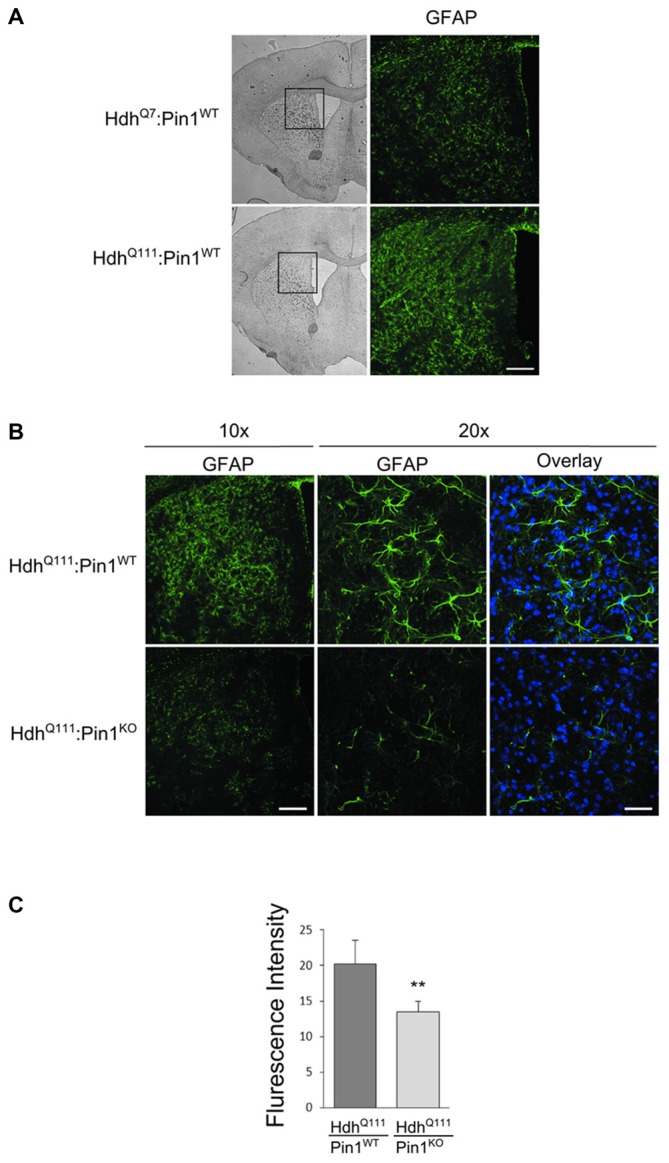
**Lack of Pin1 reduces astrocytic gliosis in striatum of aged *Hdh^Q111^* mice. (A)** Comparison of glial fibrillary acidic protein (GFAP) immunostaining between striatum of heterozygous *Hdh^Q111^* and wild-type *Hdh^Q7^* mice at 24 months of age. Left panels show representative bright-field images of mouse brain coronal sections (2.5x). The region of the striatum highlighted in the box is shown in the right panels (10x) after immunostaining with GFAP antibody. GFAP reactivity indicates reactive astrocytic gliosis in mutant compared to wild-type striatum. Scale bar, 100 μm. **(B)** Representative confocal images of GFAP staining at two different magnification (10x − 20x) of striatal sections from heterozygous *Hdh^Q111^* mice on wild-type or knock-out Pin1 genetic background. GFAP reactivity reveals reduced astrocytic gliosis in *Hdh^Q111^* mice depleted of Pin1 compared to Pin1 wild type mice. The nuclear staining with 4′,6-diamidino-2-phenylindole (DAPI) is shown in blue. (Scale bars: panel 10x = 100 μm, panel 20x = 40 μm). **(C)** Quantification (arbitrary units) of GFAP fluorescent intensity in striatum of *Hdh^Q111^* mice on wild-type or knock-out Pin1 genetic background. Histogram bars represent mean ± standard error, three sections per mouse and four mice per genotype. Data indicate significant reduction of GFAP reactivity in *Hdh^Q111^* mice lacking of Pin1 (**Two tail paired *t*-test *p* < 0.01).

Astrocytic gliosis, an important hallmark of HD pathogenesis, is recapitulated in aged *Hdh^Q111^* mice (Wheeler et al., 2002). To assess whether Pin1 may modulate the proliferation of astrocytes in *Hdh^Q111^* mice, we carried out immunofluorescence analysis using the GFAP antibody in mouse brain sections. First we compared *Hdh^Q111^* mice with *Hdh^Q7^* wild-type littermates, both on a Pin1 wild-type background, at 24 months of age. As expected, a fibrillary network intensely stained by GFAP antibody is shown in the striatum of mutant knock-in mice, but not in their wild-type counterpart (Figure 4A). Interestingly, GFAP immunoreactivity was markedly reduced in striatum of *Hdh^Q111^*:*Pin1^KO^* mice compared to littermates *Hdh^Q111^* expressing Pin1 (Figure 4B). To better assess these differences we quantified the fluorescence intensity of GFAP. As shown in Figure 4C we found a significant reduction of GFAP immunoreactivity in *Hhd^Q111^* mice lacking Pin1.

These results suggest that Pin1 is involved in astrocytosis triggered by mHtt.

## Discussion

In the quest for understanding HD pathogenesis, the use of animal models has been instrumental in evaluating the impact of manipulating the expression of a single gene or the activity of entire pathways on the hallmarks of disease progression.

*Hdh^Q111^* mice present several advantages. This genetically precise HD mouse model shows different molecular phenotypes at different ages. The main interest resides in the identification of early alterations (“early phenotypes”) that cannot be studied in human tissues. “Late phenotypes” such as the formation of NIIs and the presence of massive gliosis mimic neuro-pathological data in human post-mortem brains.

By breeding *Hdh^Q111^* knock-in mice with a knock-out line for Pin1, we assessed the effects of the lack of a functional Pin1 on selected phenotypes evident at early, mid and late stage of life of diseased mice. The selection of phenotypes was based on different objectives according to the age of the rodent.

As “early phenotypes” we specifically analyzed two events: the induction of DDR and the expression of Rrs1. mHtt is associated with DNA damage and its phosphorylation status is crucial for cellular handling of genomic stress (Anne et al., 2007). DDR leads to ATM kinase activation followed by phosphorylation of H2AX at genomic sites of double strand breaks and consequent stabilization and activation of p53. This pathway is a hallmark of the disease since H2AX-positive sites have been found in the striatum of HD human post-mortem brains and recapitulated in PC12 cells expressing a pathogenic fragment of mHtt, in HD striatal cell lines (ST*Hdh^Q111^*) and in the striatum of R6/2 mice (Anne et al., 2007; Illuzzi et al., 2009). Here we provide evidence that DDR in the striatum of *Hdh^Q111^* knock-in mice is significantly reduced in the absence of Pin1. In this context a crucial Pin1 target is p53. We previously showed that in the striatum of 12 month old *Hdh^Q111^* mice the p53 protein is increased and phosphorylated on Ser46, mimicking p53 status in HD post-mortem brains. This promotes p53/Pin1 interaction and its transcriptional activity targeted to p21^WAF^ and PUMA. p53 transcriptional property is strictly dependent on a functional Pin1 since *Hdh^Q111^* mice that are knock-out for Pin1 lack p21^WAF^ and PUMA induction (Grison et al., 2011). Apoptosis induced by mHtt is mediated at least in part by p53 since it was reduced by 50% upon p53 silencing (Bae et al., 2005). A similar effect was observed upon silencing of Pin1 in the same experimental settings. Importantly, neuronal loss observed at 24 months in Pin1 knock-out mice was not increased in *Hdh^Q111^*:*Pin1^KO^* mice suggesting that in mice devoid of Pin1 the lack of p53 activation prevents mHtt-dependent neurodegeneration (Grison et al., 2011).

We also analyzed the effects of Pin1 loss on the induction of *Rrs1*. We chose this molecular event since Rrs1 is a nucleolar protein involved in rRNA biogenesis and associated to endoplasmic reticulum stress. Both pathways are altered in several neurodegenerative diseases suggesting they might play an important role in neurodegeneration. We previously reported that *Rrs1* induction was evident for the entire first year of life (both “early” and “intermediate phenotypes”; Carnemolla et al., 2009). Here we assess that Pin1 is dispensable for *Rrs1* overexpression both at 3.5 (Figure 1D) and at 12 months (data not shown).

In summary these results suggest that Pin1 is involved in some but not all pathways altered early in *Hdh^Q111^* mice.

This observation led us to ask a crucial question: given that Pin1 participates in selected pathways triggered by mHtt, may we estimate and identify the fraction of *Hdh^Q111^* phenotypes that depend on a functional Pin1? We reasoned that the best strategy to assess this fundamental feature was to take advantage of next generation sequencing technology to carry out an unbiased transcriptome-wide approach. To study the largest number of pathways in a single experiment we choose to analyze 12 months old mice (“intermediate phenotype”). At this age mHtt can trigger a large number of alterations in the striatum while events are still probably far from being at the final irreversible disease stage.

We thus compared genes and associated biological terms that are altered in mHtt with those changing in absence of Pin1. By this approach we obtained two pieces of information. First, 10% (156/1493) of the biological terms affected by mHtt are common with those targeted by deleting Pin1 in *Hdh^Q111^* mice. This suggests that Pin1 is involved in a small portion of mHtt phenotypes. However, 44% (64/145) of the genes and 80% (156/194) of biological terms associated to loss of Pin1 are contained in the list of those targeted by mHtt, proving that the majority of pathways regulated by Pin1 in *Hdh^Q111^* mice are common with those targeted by mHtt in a Pin1 wild-type background. Half of these genes and biological terms were also changed in Pin1 knock-out mice with a wild-type Htt background.

We then showed that loss of Pin1 activity in *Hdh^Q111^* mice exerts opposite effects to those triggered by mHtt for selected pathways and functions. Among them there are “quantity/synthesis of steroids” and “synthesis/concentration of hormones”, two previously known pathways altered in HD. Concerning hormones, prolactin (PRL) and growth hormone (GH) have been found down-regulated in the plasma of HD patients (Markianos et al., 2009; Saleh et al., 2009). They are part of the hypothalamic-pituitary system that is targeted in HD affecting food intake and energy balance. Other genes decreased in *Hdh^Q111^* mice include arginine vasopressin (AVP) and proopiomelanocortin (POMC), a precursor protein of multiple peptide hormones including adrenocorticotropic hormone (ACTH) and melanocyte-stimulating hormone (MSH). While these pathways and functions are not significantly altered in Pin1 knock-out mice in a Htt wild-type background, selected genes such as PRL and GH are among the most differentially expressed genes in this comparison. Overall, these results may lead to further studies concerning the still unexplored role of Pin1 as a regulator of neuropeptides and hormone synthesis/release in health and diseased conditions.

“Wnt/β-catenin signaling”, “PCP pathway” and “Role of Wnt/GSK-3β signaling in the pathogenesis of influenza” are all changed in mHtt mice (repression) but in opposite direction when Pin1 is depleted (activation). Several evidences support the notion that Wnt/β-catenin signaling is altered in HD although contrasting data and interpretations have been proposed (Carmichael et al., 2002; Gines et al., 2003; Godin et al., 2010; Valencia et al., 2010; Dupont et al., 2012; Lim et al., 2014). In our study *Wnt9b*, *Wnt8b* and *Wnt4* levels are all decreased in the striatum of *Hdh^Q111^* mice. Furthermore, there is a significant reduction in the expression of the receptor *Fzd10* and of the transcription factor *Lef1*. Interestingly, one of the genes induced at the highest level is Wilms tumor suppressor gene (*Wt1*), a well-known regulator of the β-catenin pathway. Pin1 has been shown to regulate Wnt/β-catenin signaling pathways at least in two regulatory steps. In neuronal progenitor cells, Pin1 binds and stabilizes its substrate β-catenin and this interaction is required for appropriate neuronal differentiation (Nakamura et al., 2012). In the brain Pin1 also binds GSK-3β inhibiting its kinase activity and this is believed to play a fundamental role in promoting APP turnover (Lu et al., 1999). In the differential gene expression analysis of Pin1 knock-out mice in a Htt wild-type background all three pathways related to Wnt signaling were significantly enriched. Loss of Pin1 might thus interfere with Wnt signaling pathways at distinct regulatory steps both in wild-type and *Hdh^Q111^* mice.

As “late phenotypes” we focused our attention on the formation of NIIs and gliosis in *Hdh^Q111^* mice that both mimic neuropathological data in human post-mortem brains. It is therefore particularly interesting that both of them are modified by Pin1 loss.

NIIs are produced from the monomeric forms of mHtt through the generation of various intermediate species. The diverse toxicity of these structures is however still controversial and is discussed in several reviews (Hands and Wyttenbach, 2010; Arrasate and Finkbeiner, 2012). In brief, it has been suggested that intermediate aggregates are more toxic than inclusions. In turn these might represent a protective mechanism to sequester toxic misfolded species of mHtt that would otherwise impair cellular viability. However, in the long run, inclusions may become toxic as well, by disrupting cellular transport or sequestering protective soluble proteins.

Here we show that loss of Pin1 significantly enhances the number of NIIs specifically in the striatum of *Hdh^Q111^* mice. The mechanism that promotes the formation of inclusions in striatal neurons when Pin1 is depleted remains unclear. Pin1 exerts its activity on different substrates in different physiological and/or pathological conditions while multiple cellular pathways are involved in the aggregation process of proteins with an expanded polyglutamine tract. Indeed, conformational changes and aggregation of mHtt may be modulated by interacting partners, posttranslational modifications of the protein, as well as by alteration in the homeostasis of protein degradation systems such as UPS and autophagy. In this context, in Parkinson’s disease Pin1 has been shown to regulate alpha-synuclein aggregation by modifying the activity of the alpha-synuclein binding partner synphilin (Ryo et al., 2006). In Alzheimer’s disease Pin1 participates in APP processing and neurofibrillary tangle formation (Pastorino et al., 2006). Importantly, in Hdh knock-in mice we found the Pin1 effect visible only in striatal cells suggesting a cell-type specific activity in the very same neurons that undergo degeneration.

Proliferation of glial cells is a prominent feature of HD pathology. Reactive astrocytic gliosis is selectively detected in HD striatum starting from Grade 0 of pathology (Vonsattel et al., 1985). Several mechanisms could account for the loss of gliosis in a Pin1 knock-out background. This phenotype may depend on the established role of Pin1 in cell proliferation control and therefore be due to a cell autonomous function of Pin1 in astrocytes. However we may also speculate that this may be a secondary effect of Pin1 function in neurons. Therefore, the role of Pin1 in gliosis remains a very interesting topic to be further investigated.

It is not clear whether Pin1 effects in NIIs formation and gliosis are the effects of loss of Pin1 activity throughout the lifespan of the rodent or are the consequence of specific functions in aging mice. This observation is indeed valid for “intermediate phenotypes” as well. The use of Pin1 inhibitors in selected time windows may provide the correct answer to this important question.

In summary, we have shown that Pin1 participates in a portion of the processes at the core of HD pathogenesis *in vivo*. Further studies will evaluate the impact of these molecular phenotypes on the behavioral alterations observed in HD mice models.

## Author Contributions

EA, SG, GDS, and FP designed research; EA, SM, MM, AC, PV, PR, and SZ performed research; EA, YC, SP, CS and FP analyzed data; FM, GL and GDS contributed reagents, methods and analytic tools; SG, GDS and FP wrote the manuscript; FP supervised the entire project; EA and SM have contributed equally to this work. All authors listed, have made substantial, direct and intellectual contribution to the work, and approved it for publication.

## Funding

This work was supported by the Telethon Foundation (Grant No. GGP07185) to FP and GDS and by the Italian Ministry of Education, University and Research (FIRB Grant prot. RBAP11FRE9) to FP and SG.

## Conflict of Interest Statement

The authors declare that the research was conducted in the absence of any commercial or financial relationships that could be construed as a potential conflict of interest.

## Footnotes

^1^www.bioinformatics.babraham.ac.uk/projects/fastqc/

^2^http://www-huber.embl.de/users/anders/HTSeq

^3^www.ingenuity.com.

## Supplementary Material

Supplementary Figure S1 **Representative confocal images of EM48 positive NIIs from piriform cortex (A) and olfactory tubercle (B) of 24 months mice (genotypes as indicated).** The nuclear staining with 4′,6-diamidino-2-phenylindole (DAPI) is shown in blue. Scale bar, 20 μm.

Supplementary Table T1 **Differential gene expression in the striatum of *Hdh^Q111^* vs. *Hdh^Q7^* (*Pin1^WT^* background), *Pin1^WT^* vs. *Pin1^KO^* (*Hdh^Q111^* background) and *Pin1^WT^* vs. *Pin1^KO^* (*Hdh^Q7^* background) mice.** Significantly differentially expressed gene lists were computed using EdgeR software (see methods). For each gene table we selected the genes with adjust *p* value < 0.001 and log fold change (logFC) higher than 0.7 or smaller than −0.7. A list of 621 genes (116 increase and 505 decreased) are identified as significantly altered in *Hdh^Q111^* vs. *Hdh^Q7^* (*Pin1^WT^* background) and a list of 145 genes (48 increased and 97 decreased) are identified as differentially regulated in *Pin1^WT^* vs. *Pin1^KO^* (*Hdh^Q111^* background). By comparing *Pin1^WT^* vs. *Pin1^KO^* (*Hdh^Q7^* background) 213 genes (121 increased and 92 decreased) are identified as significantly altered.

Supplementary Table T2 **Comparison of differentially expressed gene lists.** By comparing the gene lists in Table T1 we obtained seven new list of genes. The column “n=29 Overlaps” contains the genes that are common among the three gene lists of Table T1. The column “n=85 Overlaps” contains the genes that are common between *Hdh^Q111^* vs. *Hdh^Q7^* (*Pin1^WT^*) and *Pin1^WT^* vs. *Pin1^KO^* (*Hdh^Q7^*) lists. The column “n=35 Overlaps” contains the genes that are common between *Hdh^Q111^* vs. *Hdh^Q7^* (*Pin1^WT^*) and *Pin1^WT^* vs. *Pin1^KO^* (*Hdh^Q111^*) lists. The column “n=33 Overlaps” contains the genes that are common between *Pin1^WT^* vs. *Pin1^KO^* (*Hdh^Q111^*) and *Pin1^WT^* vs. *Pin1^KO^* (*Hdh^Q7^*). The column “n=472 Specific” contains the genes that are specific for mHtt. The column “n=48 Specific” contains the genes that are specific for loss of Pin1 in a mHtt background. The column “n=66 Specific” contains the genes that are specific for loss of Pin1 in a wild-type Htt background.

Supplementary Table T3 **Functional analysis of differentially expressed genes lists.** Differentially expressed gene lists in Table T1 were analyzed for functional associations with ClusterProfiler. All mouse genes from “org.Mm.eg.db” package were used as background population. Each table reports statistical data and the gene list associated with each GO term. GO terms are based on the Gene Ontology Consortium available at: http://www.geneontology.org/.

Supplementary Table T4 **Comparison of functional analysis lists.** By comparing the functional analysis lists in Table T3 we obtained seven new lists: 1) “n=75 Overlaps” contains 75 GO terms that are common among the three lists of Table T3; 2) “n=135 Overlaps” contains 135 GO terms that are common between *Hdh^Q111^* vs. *Hdh^Q7^* (*Pin1^WT^*) and *Pin1^WT^* vs. *Pin1^KO^* (*Hdh^Q7^*) lists; 3) “n=81 Overlaps” contains 135 GO terms that are common between *Hdh^Q111^* vs. *Hdh^Q7^* (*Pin1^WT^*) and *Pin1^WT^* vs. *Pin1^KO^* (*Hdh^Q111^*) lists; 4) “n=3 Overlaps” contains 3 GO terms that are common between *Pin1^WT^* vs. *Pin1^KO^* (*Hdh^Q111^*) and *Pin1^WT^* vs. *Pin1^KO^* (*Hdh^Q7^*); 5) “n=1202 Specific” contains contains 1202 GO terms specific for *Hdh^Q111^* vs. *Hdh^Q7^* (*Pin1^WT^*); 6) “n=35 Specific” contains 35 GO terms specific for *Pin1^WT^* vs. *Pin1^KO^* (*Hdh^Q111^*); 7) “n=29 Specific” contains 29 GO terms specific for *Pin1^WT^* vs. *Pin1^KO^* (*Hdh^Q7^*).

Supplementary Table T5 **Activation and repression of biological terms with Ingenuity Pathway Analysis.** This table shows the predicted activation and repression of different biological terms obtained from Table T1 using IPA. For each table, the gene list associated to each pathway or function is reported.
