# Supplementary figures and images for: Effects of Pin1 Loss in HdhQ111 Knock-in Mice
Source: Front Cell Neurosci. 2016 May 2;10:110. doi: 10.3389/fncel.2016.00110 (PMC4852193; doi:10.3389/fncel.2016.00110)

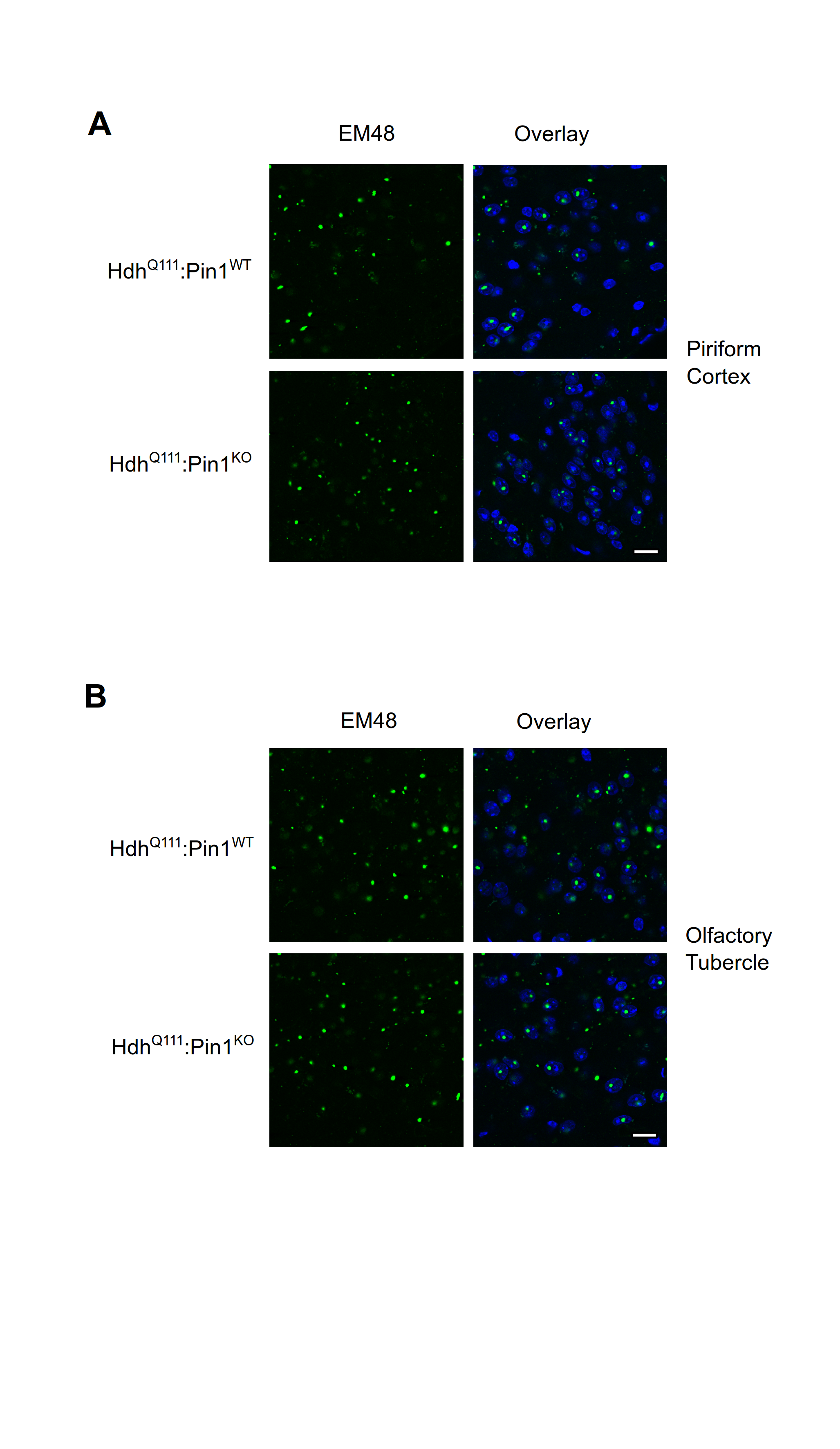

Supplement: Supplementary Figure S1 — Representative confocal images of EM48 positive NIIs from piriform cortex (A) and olfactory tubercle (B) of 24 months mice (genotypes as indicated). The nuclear staining with 4′,6-diamidino-2-phenylindole (DAPI) is shown in blue. Scale bar, 20 μm. [file Image_1.tif]
